# Kupffer Cell-mediated Recruitment of Dendritic Cells to the Liver Crucial for a Host Defense

**DOI:** 10.1080/1044667031000137610

**Published:** 2002-09

**Authors:** Kenjiro Matsuno, Hisayuki Nomiyama, Hiroyuki Yoneyama, Ryosuke Uwatoku

**Affiliations:** 1 Department of Anatomy (Macro) and SORST Dokkyo University School of Medicine Mibu Tochigi 321-0293 Japan; 2 Department of Biochemistry I Kumamoto University School of Medicine Kumamoto 860-0811 Japan; 3 Department of Molecular Preventive Medicine School of Medicine and SORST The University of Tokyo Tokyo 113-0033 Japan

## Abstract

Tissue recruitment of dendritic cells (DCs) is essential for antigen presentation. When latex particulates were injected intravenously into rats, DC precursors were recruited to the liver. *Propionibacterium acnes* also induced the recruitment of definite mouse DC precursors. These DCs initially showed a selective binding to Kupffer cells. In the Kupffer cell-depleted rats, DCs could neither be recruited to the liver nor adhere to sinusoidal walls. Pretreatment with varied monosaccharides *in vitro* showed that sugar residues consisting of N-acetylgalactosamine were necessary for this binding. Mouse DC precursors had CC-chemokine receptor 1 and 5, while granulama tissues and rat Kupffer cells expressed the corresponding chemokine, macrophage inflammatory protein-1α. Recruited DC precursors phagocytosed latex or bacteria and some of them soon translocated to hepatic nodes and induced the immune response there. We conclude that after invasion of pathogens, Kupffer cells not only scavenge them but also recruit DCs/DC precursors via chemokine- and N-acetylgalactosamine-mediated interactions. The accelerated DC traffic and the presence of blood-lymph translocation would induce rapid and efficient immune responses and thus contribute to the local defense to antigens within liver tissues as well as systemic defense to blood-borne antigens.


*Developmental Immunology*, September 2002, Vol. 9 (3), pp. 143–149

Taylor & Francis

Taylor & Francis Group

### Kupffer Cell-mediated Recruitment of Dendritic Cells to the Liver Crucial for a Host Defense*

KENJIRO MATSUNO[a,†], HISAYUKI NOMIYAMA[b], HIROYUKI YONEYAMA[c] and RYOSUKE UWATOKU[a]

[a]*Department of Anatomy (Macro) and SORST, Dokkyo University, School of Medicine, Mibu, Tochigi 321-0293, Japan;* [b]*Department of Biochemistry I, Kumamoto University, School of Medicine, Kumamoto 860-0811, Japan;* [c]*Department of Molecular Preventive Medicine, School of Medicine and SORST, The University of Tokyo, Tokyo 113-0033, Japan*

Tissue recruitment of dendritic cells (DCs) is essential for antigen presentation. When latex particulates were injected intravenously into rats, DC precursors were recruited to the liver. *Propionibacterium acnes* also induced the recruitment of definite mouse DC precursors. These DCs initially showed a selective binding to Kupffer cells. In the Kupffer cell-depleted rats, DCs could neither be recruited to the liver nor adhere to sinusoidal walls. Pretreatment with varied monosaccharides *in vitro* showed that sugar residues consisting of N-acetylgalactosamine were necessary for this binding. Mouse DC precursors had CC-chemokine receptor 1 and 5, while granulama tissues and rat Kupffer cells expressed the corresponding chemokine, macrophage inflammatory protein-1α. Recruited DC precursors phagocytosed latex or bacteria and some of them soon translocated to hepatic nodes and induced the immune response there. We conclude that after invasion of pathogens, Kupffer cells not only scavenge them but also recruit DCs/DC precursors via chemokine- and N-acetylgalactosamine-mediated interactions. The accelerated DC traffic and the presence of blood-lymph translocation would induce rapid and efficient immune responses and thus contribute to the local defense to antigens within liver tissues as well as systemic defense to blood-borne antigens.

*Keywords*: Dendritic cell; Cell recruitment; Liver; Kupffer cell; MIP-1α; N-acetylgalactosamine

### INTRODUCTION

Dendritic cells (DCs) are bone marrow derived cells that function as professional antigen presenting cells. DCs comprise five maturational stages; progenitors in the bone marrow, precursors in the blood, immature sentinel DCs in peripheral tissues, antigen-transporting DCs in the afferent lymph and the blood and mature antigen-presenting DCs in lymphoid tissues. The ability of DCs to be recruited to peripheral tissues is their essential function for antigen presentation. The liver is indispensable for a host defense. However, recruitment of DCs to the liver tissues is still not well characterized both in cellular and molecular levels (Matsuno and Ezaki, 2000).

Intravenously (i.v.) injected foreign bodies are mostly scavenged by Kupffer cells (KCs). In this occasion, we found that particle-laden cells having a DC marker successively appeared in the sinusoidal area, and then in the portal area of the rat liver after i.v. injection of latex microspheres (Matsuno *et al.*, 1996). These particle-laden cells were speculated to be DC precursors with a phagocytic activity, which were recruited to the liver in response to particulate injection (Austyn, 1996). Eventually, a portion of the particle-laden DCs entered the lymphatic capillaries in the portal area and finally accumulated in the hepatic lymph nodes (LNs). This unique migration pattern was designated as the hepatic sinusoids-lymph translocation (Kudo *et al.*, 1997). DCs at the antigen-transporting stage emigrate from the peripheral tissues and enter mostly the afferent lymph or partly the blood (Austyn, 1996). Lymph DCs at this stage also readily underwent the sinusoids-lymph translocation when i.v. injected into normal rats, indicating their potential to be recruited to the liver. Furthermore *in vitro*, these cells showed a selective binding to KCs on liver cryosections by an *in situ* cell binding assay (Kudo *et al.*, 1997).

These findings suggest that recruitment of the rat DC lineage to the liver may be critically regulated by interactions between DCs and KCs. For their interactions, an involvement of certain adhesion molecules as well as chemokines is highly suspected. The aim of this study is therefore, to elucidate the actual cellular and molecular mechanisms for DC recruitment to the liver.

*Presented at the Proceedings of the 4th Germinal Center Conference, Groningen, The Netherlands, June 2002.

†Corresponding author. Tel.: +81-282-86-6238. Fax: +81-282-96-6229. E-mail: kenjiro@dokkyomed.ac.jp

ISSN 1044-6672 print © 2002 Taylor & Francis Ltd
DOI: 10.1080/1044667031000137610

### MATERIALS AND METHODS

### Animals and Cells

Inbred DA rats ($RT1^a$), Lewis rats ($RT1^l$) and Wistar rats were used for DC transfer and cell binding assay. Hepatointestinal lymph DC were collected and isolated from male DA rats as described previously (Uwatoku *et al.*, 2001). The yield of DCs was usually in the range of $1-3 \times 10^6$/rat/overnight collection, with the purity of 60–80% and viability of more than 95%. KCs from Wistar rats were also isolated by enzymal digestion and centrifugal elutriation with a purity of $>95\%$ (Kristensen *et al.*, 2000). Specific pathogen-free female BALB/c mice were injected with heat-killed *P. acnes* (ATCC 11828; 1 mg/μl in PBS) via the tail vein, which resulted in hepatic granuloma formation by 7 days (Yoneyama *et al.*, 1998). Handling and the care of animals were approved by the Kumamoto University Animal Experiment Committee and in accordance with University Kumamoto's Regulation for Animal Experiments and Japanese Governmental Law (No. 105).

### Antibodies and Reagents

Mouse mAbs for rat determinants, being specific for a polymorphic major histocompatibility complex class II antigen (MHC II) of DA rat (anti-$RT1.B^a$, OX76), DC subpopulation (OX62) and rat tissue macrophages including KCs (ED2) were obtained from Serotec Ltd. (Kidlington, Oxford, UK). A rabbit polyclonal antibody against mouse type IV collagen to outline sinusoidal vascular linings was obtained from Cosmo Bio (Tokyo, Japan). The following anti-mouse mAbs were used for the *P. acnes* study. CD8α (53-6.7), CD11c (HL3), B220 (RA3-6B2), CD86 (GL-1), F4/80 (CI:A3-1), DEC-205 (NLDC-145), MHC class II (ER-TR3) and CD11c (N418) (Serotec).

As secondary antibodies, an alkaline phosphatase (ALP)-labeled goat Ig to mouse Ig (Sigma Chemical Co.), an ALP-labeled anti-rat or hamster IgG (Jackson ImmunoResearch), a horseradish peroxidase (HRP)-labeled rabbit Ig to mouse Ig (DAKO Corp., Carpinteria, CA), an HRP-labeled goat $F(ab')_2$ to rabbit Ig (55693, Cappel, Aurora, OH) were used. Substrates were a Vector Blue kit for ALP (blue reaction product, Vector Laboratories Inc., Burlingame, CA), a New Fuchsin kit for ALP (red, DAKO), and diaminobenzidine for HRP (brown, WAKO Pure Chemical Industries, Osaka, Japan). Monosaccharides used for the cell binding assay (Nacalai Tesque, Inc., Kyoto, Japan) were D-glucose, D-fucose, L-fucose, D-galactose, D-mannose, N-acetylgalactosamine (GalNAc) and N-acetylglucosamine.

### DC Precursor Recruitment

It is a current opinion that DC precursors are non-proliferating and produced by a replication of DC progenitors mostly in the bone marrow. Since latex-laden $OX62^+$ putative rat DC precursors appeared in the sinusoidal area after latex injection (Matsuno *et al.*, 1996), we speculated that at least a portion of DC precursors might bear OX62 marker from the beginning of their recruitment. If $OX62^+$ cells are labeled by a short term BrdU feeding before latex injection, one could confirm that these cells are newly produced precursors, i.e. daughter cells of DC progenitors. Accordingly, proliferating cells of female DA rats were labeled *in vivo* with BrdU (Sigma Chemical Co.) delivered in drinking water ad libitum at a concentration of 0.8 mg/ml for 2 days. Immediately after cessation of the BrdU feeding, rats received an i.v. injection of latex particles (Nile red FluoSpheres, 0.5 ml/rat, Molecular Probes, Inc.) and the liver cryosections were immunostained at 6 and 12 h.

Since $CD11c^+$ cells largely increased in number in the mice liver after *P. acnes* injection, we speculated that these cells might correspond to mice DC precursors during the liver infection. Accordingly, phenotype and kinetics of these cells in the blood and liver nonparenchymal cell fractions were examined.

### Kinetics of Transferred DCs *In Vivo*

To examine DC recruitment to the liver *in vivo*, DCs labeled with 5-carboxyfluorescein succinimidyl ester (CFSE, Molecular Probes) were i.v. transferred and chased intravitally through confocal laser-scan digital video microfluorography (Uwatoku *et al.*, 2001). To know a role of KCs for the DC recruitment, KCs were depleted by i.v. injection of liposomes containing dichloromethylene diphosphonate (650 mM, 1 ml/rat), 24 h before DC transfer ($N = 3$). This procedure induced selective apoptosis of KCs (Naito *et al.*, 1996) but did not affect hepatocyte function.

### *In Situ* Cell Binding Assay

To know tissue and cellular specificity of DC–KC binding, the binding assay of DCs to tissue sections was performed as described (Kudo *et al.*, 1997). For sugar inhibition test, DCs were preincubated with either 25, 50 or 100 mM of monosaccharides in Dulbecco's modified Eagle medium with HEPES for 30 min at 37°C, then the cell binding assay was performed.

### cDNA Array Hybridization of Isolated Rat KCs

Isolated KCs of Wistar rats were cultured for 3 h and 16 h in Dulbecco's modified Eagle medium containing *E. coli* B:011 lipopolysaccharide (500 ng/ml). Clontech Atlas Rat arrays (cat. no. 7854-1) displaying 1176 rat known genes were hybridized to $^{33}P$-labeled cDNA probes prepared from these cells. Probe labeling and hybridizations were performed according to the manufacturer's instructions. The filters were exposed to films overnight at $-70$°C. The signals were identified by phosphoimaging and quantified using the AtlasImage software version 2.0 (Clontech).

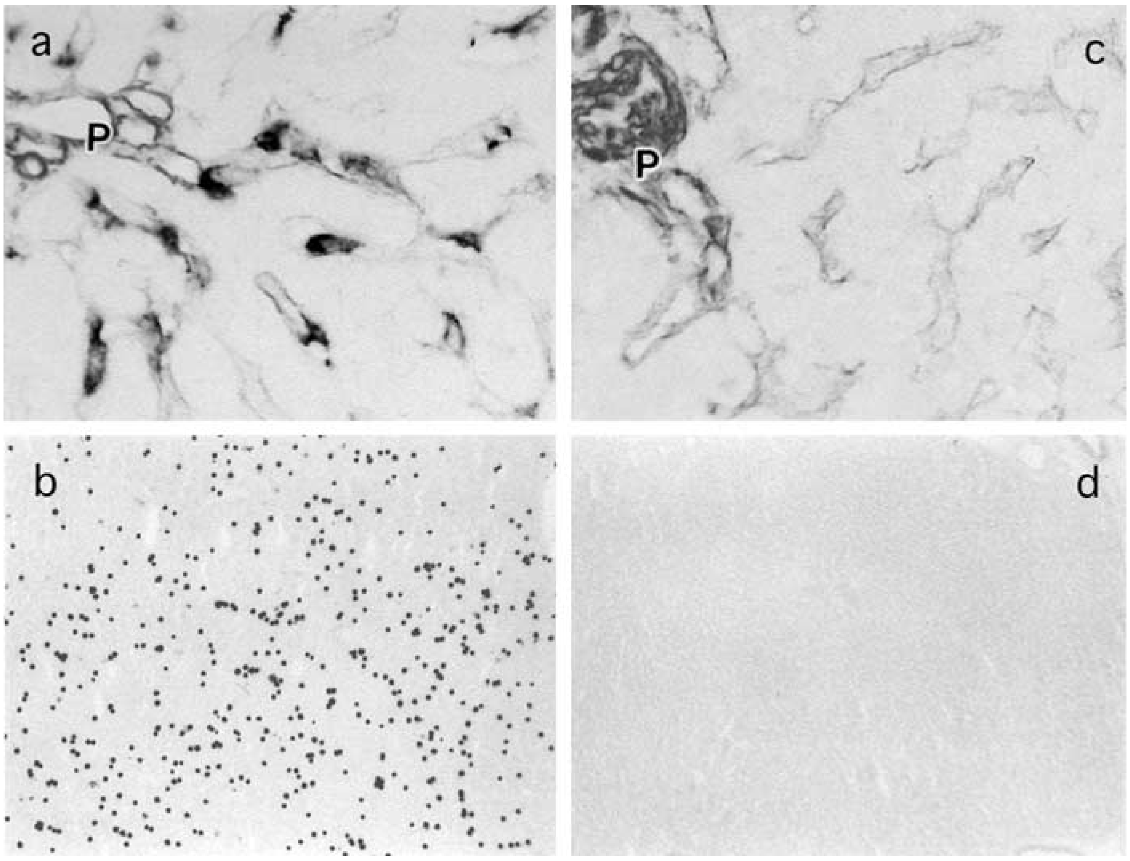

FIGURE 1 A liver section of normal (a, b) and KC-depleted (c, d) rats. a and c: immunostaining for KCs (ED2, blue) and sinusoidal linings (type IV collagen, brown). b and d: *in situ* cell binding assay of DCs to liver section. P, portal area. Scale bars 20 μm (a, c) and 200 μm (b, d).

The threshold values were set to 5.0 (ratio threshold) and 100 (difference threshold) for up-regulated genes and to 5.0 (ratio threshold) and 50 (difference threshold) for down-regulated genes.

### RT-PCR in Mice *P. acnes* Model

Total RNA was isolated from liver specimens and $2 \times 10^5$ sorted blood or liver $CD11c^+$ cells using RNAzol$^B$ (BIOTEX LAB.) and reverse transcribed (Yoneyama *et al.*, 2001). Thereafter, cDNA was amplified using the ABI 7700 sequence detector system (PE Applied Biosystems) with a set of primers and probes corresponding to macrophage inflammatory protein-1α (MIP-1α), secondary lymphoid organ chemokine (SLC), CC-chemokine receptor 1 (CCR1), CCR5, CCR7 and glyceraldehyde-3-phosphate dehydrogenase. The primers for SLC were: 5′-GGCTATAGGAAGCAAGAACCAAGT-3′ and 5′-TCAGGCTTAGAGTGCTTCCG-3′. The probes were as follows: 5′-CCCATCCCGGCAATCCTGTTCTTA-3′ for SLC and 5′-ATCACCATCCAAGTGGCCCAGATGGT-3′ for CCR7.

### Chemotaxis Assay in Mice *P. acnes* Model

Freshly isolated peripheral blood leukocytes (PBLs) and NPCs ($8 \times 10^5$/120 μl in RPMI 1640 plus 0.25 % bovine serum albumin) obtained from *P. acnes*-primed mice at day 7 were loaded into murine type IV collagen-coated transwells (Becton Dickinson, 3 μm pore size), which were placed in a 24-well tissue culture plate containing 500 μl of the medium supplemented with or without 100 ng/ml of MIP-1α, MDC, IP-10 (R and D systems) or SLC (Yoneyama *et al.*, 2001). After an incubation period of 4 h at 37°C, cells in the bottom of each well were collected and counted with a hemocytometer. After immunostaining for CD11c, the absolute number of migrated $CD11c^+$ cells was determined by multiplying the total migrated cell number by the fraction of $CD11c^+$ population.

### RESULTS

### DC Precursor Recruitment

After i.v. injection of latex microspheres into rats which had been fed with BrdU for 2 days, $BrdU^+OX62^+$ cells significantly increased in number by 12 h. $BrdU^+OX62^+$ cells are small round cells, and their majority ingested latex particles (Uwatoku *et al.*, 2001). These cells soon appeared in the portal area and showed the same kinetics as that of latex-laden DCs described previously (Matsuno *et al.*, 1996). The results indicate that the recruitment of rat

DC precursors significantly occurred in the sinusoidal area 12 h after latex injection.

Mice DC precursors (MHCII$^-$DEC205$^-$CD86$^-$CD8$^-$ F4/80$^-$CD11c$^+$) appear in the blood from 1 h after *P. acnes* i.v injection. DC precursors capture *P. acnes* antigens, show self-replicating activity and mature (MHCII$^+$DEC205$^+$CD86$^+$CD8$^-$F4/80$^-$CD11c$^+$) within granuloma tissues (Yoneyama *et al.*, 2001).

### *In Vivo* Binding of DCs to KCs

A video clip showing *in vivo* kinetics of CFSE-labeled DCs in hepatic microcirculation is available at our web site (http://macro.dokkyomed.ac.jp/gastro/). Immediately after the injection into circulation, the fluorescent DCs were observed to come up through terminal portal venules and displayed a sudden arrest in sinusoids in periportal regions. On the other hand, in rats undergoing the KC-depleting procedure, the majority of fluorescent cells exhibited rolling and/or free-flowing along sinusoids without any notable signs of arresting behavior. The arrest of DCs in sinusoids appeared to be an event occurring specifically in these cells, since other immune cells, such as spleen lymphocytes, injected into circulation did not exhibit any changes of sinusoidal arrest (data not shown). Consequently, at 30 min after the injection, DCs were captured preferentially in sinusoids of periportal regions in the control rats, while displaying virtually no sinusoidal binding in the KC-depleted animals. A subpopulation of DCs adhered to sinusoids exhibited extravasation and migration into the space of Disse within the initial 1 h after their injection. Adjustment of Z-axis focusing in the laser confocal system allowed us to show that a DC was out of focus from sinusoidal plane indicating extravasation.

### *In Situ* Cell Binding Assay

DCs bound to the liver sections with a highly specific manner in not only allogeneic but also syngeneic rats. On the liver sections, DCs showed a selective binding to KCs. In fact, DC did not bind to KC-depleted liver sections at all (Fig. 1). In contrast, much fewer number of DCs bound to most other tissues examined. The pretreatment of DCs with varied monosaccharide revealed that GalNAc, D-fucose, D-galactose and N-acetylglucosamine inhibited the DC binding to the liver sections. GalNAc showed a stronger inhibition than all other sugars at 25 mM (Uwatoku *et al.*, 2001).

### mRNA Expression Profile of LPS-Treated Rat KCs

Figure 2 shows the scanned hybridized arrays with differentially expressed genes and housekeeping genes. Examples of up- and down-regulated genes are shown in Fig. 3. The expression of certain genes was significantly increased in KCs after 3 h incubation with LPS, compared with cultures without LPS stimulation (Fig. 3A). The up-regulated genes were clustered according to gene function. A first set was represented by proinflammatory cytokines and chemokines, including CC chemokine MIP-1α (51.4- and 18.0-fold at 3 and 16 h, respectively), CXC chemokine IP-10 (157.7- and 5.6-fold at 3 and 16 h, respectively), interleukin-1β (IL-1β) (26.9- and 15.5-fold at 3 and 16 h, respectively), tumor necrosis factor-α (TNFα) (169.0- and 2.6-fold at 3 and 16 h, respectively), and CXC chemokine MIP-2 (67.7- and 45.2-fold at 3 and 16 h, respectively). Among them, MIP-1α gene expression far exceeded the other transcripts. A second set of LPS-inducible transcripts is related to regulators of tissue invasion and includes inducible nitric oxide synthase (iNOS) (167.0- and 191.0-fold at 3 and 16 h, respectively)

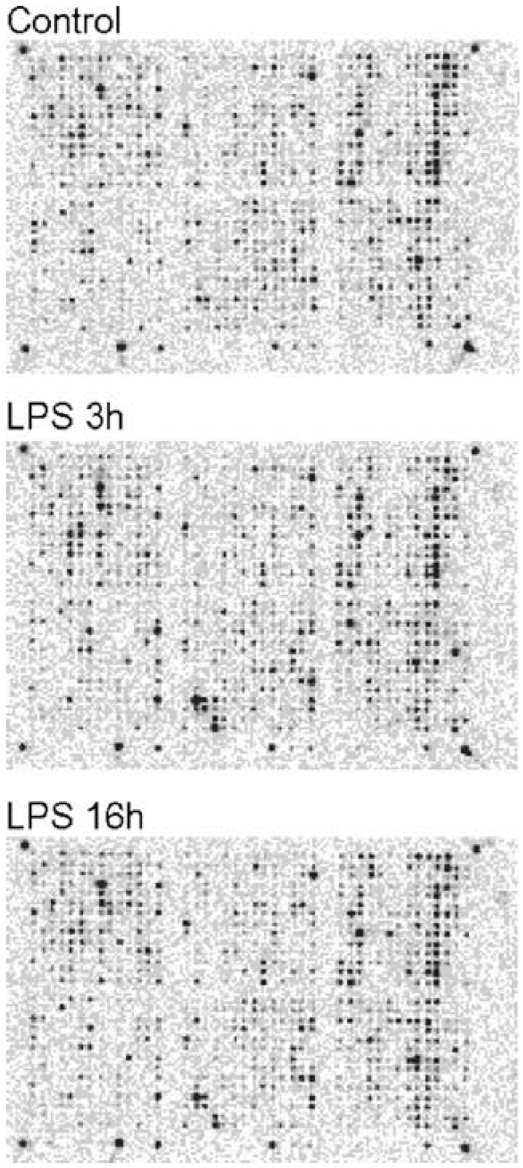

FIGURE 2 cDNA hybridization. Atlas filter arrays were hybridized to $^{32}$P-labeled cDNA probes prepared from rat KCs nonstimulated or stimulated with LPS for 3 and 16 h. The filters were exposed overnight to X-ray films and developed.

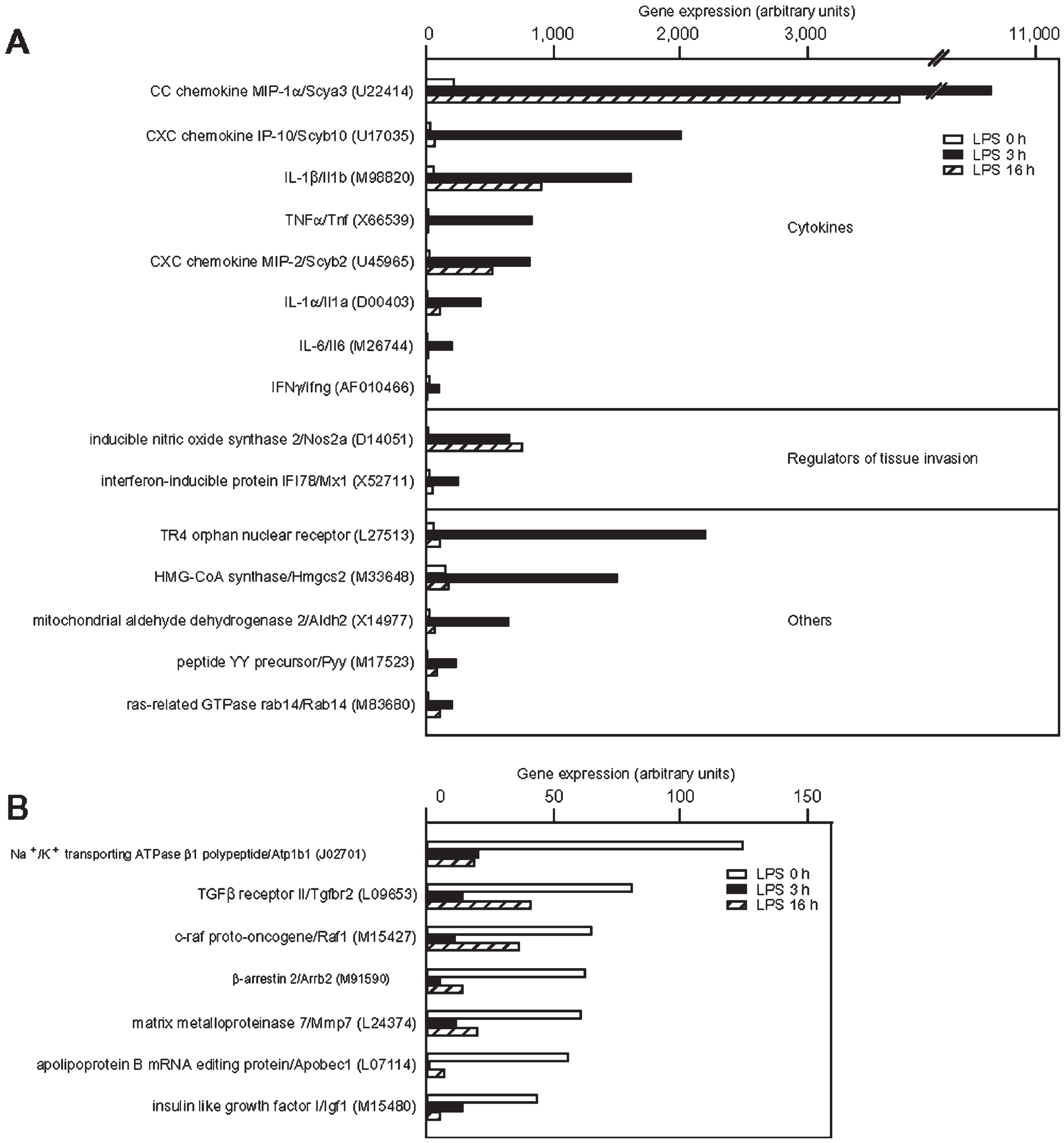

FIGURE 3 Genes differentially expressed in LPS-stimulated KCs. Genes up-regulated (A) and down-regulated (B) in LPS-treated KCs are shown. Rat KCs were stimulated with 500 ng/ml LPS for indicated time. Poly $(A)^+$ RNA was prepared, and gene expression profiles in non-stimulated and LPS-stimulated KCs were analyzed by cDNA arrays. LPS-responsive genes are shown with the corresponding gene symbols and GenBank accession numbers. Expression levels at time zero (□) and after 3 h (■) and 16 h (▨) of treatment with LPS are shown.

and interferon-inducible protein IFI78 (also known as myxovirus (influenza) resistance) (12.7- and 3.6-fold at 3 and 16 h, respectively). Other functional sets included nuclear proteins (e.g. orphan nuclear receptor TR4 of steroid receptor superfamily), metabolic enzymes (e.g. mitochondrial aldehyde dehydrogenase 2) and signal transducers (e.g. ras-related GTPase rab14). Expression of the up-regulated genes was down-modulated at 16 h after stimulation except the iNOS gene, which was expressed at the same level at 3 and 16 h.

Compared to the up-regulated genes, inhibition of gene expression by LPS in KCs is rather mild (Fig. 3B). The down-regulated genes include Na+/K+ transporting ATPase b1 polypeptide, TGF-β receptor II and c-raf proto-oncogene.

### Role of Chemokines on Kinetics of Mice DC Precursors

Blood $CD11c^+$ DC precursors predominantly expressed CCR1 and CCR5 and showed a significant chemotactic response toward MIP-1α. MIP-1α was weakly expressed in the sinusoidal endothelial cells and KCs in untreated liver, but was produced at high levels in the sinusoidal granulomas of inflamed liver (Yoneyama *et al.*, 2001).

### DISCUSSION

In this report, we have examined the mechanisms for recruitment of the DC lineage to the rat and mouse livers both *in vivo* and *in vitro*. We revealed that DCs not only at the antigen-transporting stage but also at the precursor stage could be recruited to the liver and that their majority showed a selective binding to KCs in the sinusoidal area. In the KC-depleted rats, on the other hand, DCs could neither be recruited to the liver nor adhere to sinusoidal walls. For the DC–KC binding, GalNAc sugar residue-receptor interactions were mainly responsible. In mice, DC precursors also quickly appear in the blood from 1 h after *P. acnes* i.v. injection, capture *P. acnes* antigens, show self-replicating activity and mature within granuloma tissues. These precursors are CCR1/5$^+$ and selectively chemoattracted by MIP-1α, which may be produced by KCs in the granuloma tissues. Since isolated KCs showed a very high gene expression of MIP-1α after LPS stimulation in rat system, we suggest that activated KCs secrete MIP-1α which induce DC recruitment from the circulation in both rats and mice.

The recruitment of DC lineage to the liver may be closely related to a host defense (see a diagram, Fig. 4). A predominant accumulation of i.v. transferred DCs in the liver not only in rats (Kudo *et al.*, 1997) but also in mice (Kupiec-Weglinski *et al.*, 1988) and human (Mackensen *et al.*, 1999) suggest that this recruitment is a regular migration pathway for DCs in the blood. In many species, blood-borne particulate antigens are mostly scavenged by KCs and eventually accumulate in the liver. KCs are activated by phagocytosis and secrete chemokines MIP-1α that may attract DCs/ DC precursors from other tissues via blood. DCs that have migrated to the liver initially bind to KCs by which these cells can adhere to the sinusoidal wall and then extravasate into the space of Disse, achieving their recruitment. Recruited DCs may capture antigens in the blood during their migration and induce local immune responses. Some DCs further translocate to the regional hepatic LNs and transport antigenic information. In mice *P. acnes* model, DC precursors further performed migration to the portal area and hepatic LNs and induced T-cell proliferative responses (Yoneyama *et al.*, 2001), indicating the importance of this recruitment in host defense to bacterial infection. DCs at the antigen-transporting stage also transport antigenic information from non-lymphoid organs to the hepatic nodes (Saiki *et al.*, 2001) in addition

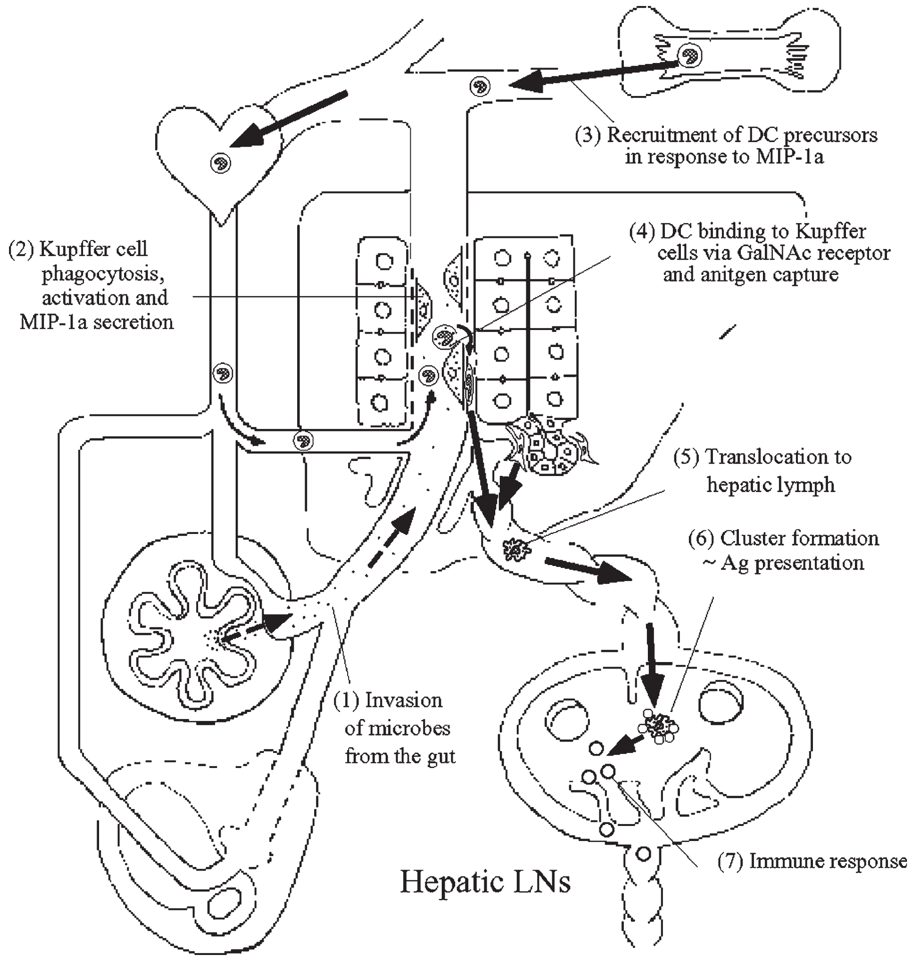

FIGURE 4 Diagram illustrating the proposed molecular mechanisms for the DC recruitment to the liver and eventual immune responses in the regional hepatic nodes as one of the most efficient and powerful tools for the host defense.

to the established pathway to the spleen. In conclusion, we suggest that the machinery for the hepatic DC recruitment would provide rapid and efficient immune responses and thus contribute to the local defense to antigens within liver tissues as well as systemic defense to blood-borne antigens from the gut or other peripheral tissues. Further analysis for the DC-KC interactions will facilitate understanding of DC lectins, chemokine network and cross-talk between both cells, which may provide useful information for therapy of liver diseases.

### *References*

Austyn, J.M. (1996) "New insights into the mobilization and phagocytic activity of dendritic cells [comment]", *J. Exp. Med.* **183**, 1287–1292.

Kristensen, D.B., Kawada, N., Imamura, K., Miyamoto, Y., Tateno, C., Seki, S., Kuroki, T. and Yoshizato, K. (2000) "Proteome analysis of rat hepatic stellate cells", *Hepatology* **32**, 268–277.

Kudo, S., Matsuno, K., Ezaki, T. and Ogawa, M. (1997) "A novel migration pathway for rat dendritic cells from the blood: hepatic sinusoids-lymph translocation", *J. Exp. Med.* **185**, 777–784.

Kupiec-Weglinski, J.W., Austyn, J.M. and Morris, P.J. (1988) "Migration patterns of dendritic cells in the mouse. Traffic from the blood, and T cell-dependent and -independent entry to lymphoid tissues", *J. Exp. Med.* **167**, 632–645.

Mackensen, A., Krause, T., Blum, U., Uhrmeister, P., Mertelsmann, R. and Lindemann, A. (1999) "Homing of intravenously and intralymphatically injected human dendritic cells generated *in vitro* from CD34+ hematopoietic progenitor cells", *Cancer Immunol. Immunother.* **48**, 118–122.

Matsuno, K. and Ezaki, T. (2000) "Dendritic cell dynamics in the liver and hepatic lymph", *Int. Rev. Cytol.* **197**, 83–136.

Matsuno, K., Ezaki, T., Kudo, S. and Uehara, Y. (1996) "A life stage of particle-laden rat dendritic cells *in vivo*: their terminal division, active phagocytosis and translocation from the liver to the draining lymph", *J. Exp. Med.* **183**, 1865–1878.

Naito, M., Nagai, H., Kawano, S., Umezu, H., Zhu, H., Moriyama, H., Yamamoto, T., Takatsuka, H. and Takei, Y. (1996) "Liposome-encapsulated dichloromethylene diphosphonate induces macrophage apoptosis *in vivo* and *in vitro*", *J. Leukoc. Biol.* **60**, 337–344.

Saiki, T., Ezaki, T., Ogawa, M. and Matsuno, K. (2001) "Trafficking of host- and donor-derived dendritic cells in the rat cardiac transplantation: allosensitization in the spleen and hepatic nodes", *Transplantation* **71**, 1806–1815.

Uwatoku, R., Suematsu, M., Ezaki, T., Saiki, T., Tsuiji, T., Irimura, T., Kawada, N., Suganuma, T., Naito, M., Ando, M. and Matsuno, K. (2001) "Kupffer cell-mediated recruitment of rat dendritic cells to the liver: roles of N-acetylgalactosamine-specific sugar receptors", *Gastroenterology* **121**, 1460–1472.

Yoneyama, H., Harada, A., Imai, T., Baba, M., Yoshie, O., Zhang, Y., Higashi, H., Murai, M., Asakura, H. and Matsushima, K. (1998) "Pivotal role of TARC, a CC chemokine, in bacteria-induced fulminant hepatic failure in mice", *J. Clin. Invest.* **102**, 1933–1941.

Yoneyama, H., Matsuno, K., Zhang, Y., Murai, M., Itakura, M., Ishikawa, S., Hasegawa, G., Naito, M., Asakura, H. and Matsushima, K. (2001) "Regulation by chemokines of circulating dendritic cell precursors, and the formation of portal tract-associated lymphoid tissue, in a granulomatous liver disease", *J. Exp. Med.* **193**, 35–49.

